# Effects of tributyrin and alanyl-glutamine dipeptide on intestinal health of largemouth bass (*Micropterus salmoides*) fed with high soybean meal diet

**DOI:** 10.3389/fimmu.2023.1140678

**Published:** 2023-05-17

**Authors:** Jianhua Zhao, Xin Yang, Zongsheng Qiu, Rongfei Zhang, Hong Xu, Ting Wang

**Affiliations:** ^1^College of Life Science, Huzhou University, Huzhou, China; ^2^National Local Joint Engineering Laboratory of Aquatic Animal Genetic Breeding and Nutrition, Huzhou, China; ^3^Zhejiang Provincial Key Laboratory of Aquatic Resources Conservation and Development, Huzhou, China; ^4^School of Foreign Languages, Huzhou University, Huzhou, China

**Keywords:** tributyrin, alanyl-glutamine, intestinal morphology, inflammation, soybean meal, largemouth bass

## Abstract

To investigate the effects of dietary tributyrin (TB) and alanyl-glutamine (AGn) on the intestinal health of largemouth bass (*Micropterus salmoides*) fed with high-level soybean meal (SM) diet, six isonitrogenous (41.36%) and isolipidic (10.25%) diets were formulated and fed to largemouth bass (initial body weight 25.5 ± 0.5g) for 8 weeks. The two control diets contained 34.8% peanut meal (PM) and 41.3% SM, while the other four experimental diets supplemented TB at 0.1% (TB0.1), 0.2% (TB0.2) and AGn at 1% (AGn1), 2% (AGn2) in SM, respectively. The results showed that there were no significant differences in weight gain, survival rate, and hepatosomatic index among all groups (*P*>0.05), while feed coefficient rate in AGn1, AGn2 and TB0.2 groups was significantly lower than that in SM group (*P*< 0.05). Compared with the PM group, the intestinal inflammation of largemouth bass in SM group were obvious, accompanied by the damage of intestinal structure, the decrease of digestive enzyme activity, and the up-regulation of proinflammatory cytokines. Compared with the SM group, the activities of intestinal trypsin, lipase and foregut amylase in TB and AGn groups increased significantly (*P*<0.05), and the gene expression levels of acetyl-CoA carboxylase (ACC), caspase-3, caspase-8, caspase-9, tumor necrosis factor alpha (TNF-α), and interleukin-1 beta (IL-1β) were down-regulated, while the gene expression levels of target of rapamycin (TOR) and eIF4E-binding protein (4E-BP) were up-regulated in all experimental groups (*P*<0.05). It can be concluded that supplementation of 1%-2% AGn and 0.1%-0.2% TB can alleviate enteritis caused by high-level soybean meal, and the recommend level is 2% AGn and 0.2% TB.

## 1 Introduction

Fish meal is widely used in aquatic animal feed. However, due to the limitation of natural resources and the climate change, the shortage of fish meal supply is increasing. In order to improve the sustainability and profitability of aquaculture, plant protein was paid attention in recent years. Soybean meal (SM) has become one of the main protein sources in aquatic feed because of its high protein content, relatively balanced amino acid composition and reasonable price. However, SM contains some anti-nutritional factors including trypsin inhibitors, antigen proteins, lectins, tannins, saponins and alkaloids (1–3). Previous studies on Atlantic salmon (*Salmo salar*) (4, 5), zebrafish (*Danio rerio*) (6), rice field eel (*Monopterus albus*) (7), Japanese seabass (*Lateolabrax maculatus*) (8), largemouth bass (9) and Mirror carp (*Cyprinus carpio*) (10) have shown that, excessive use of SM in feed can cause intestinal inflammation and injury, intestinal digestion and absorption dysfunction, and ultimately reduce growth performance (11, 12), especially in carnivorous fish.

Intestine is an important organ of fish, involved in digestion, absorption and immunity (13). Intestinal mucosal barrier dysfunction is a prerequisite for intestinal diseases (14). In this case, it is necessary to adopt nutritional strategies such as supplementing some functional feed additives to alleviate intestinal inflammation and disease in animals. Both glutamine and butyric acid play important roles in providing energy to the intestine and improving intestinal immunity (15, 16). Glutamine is a major fuel source for rapidly dividing cells such as intestinal epithelial cells, macrophages, and lymphocytes in the intestine, suggesting that glutamine can maintain intestinal integrity and activate the immune system (15). However, due to the low solubility and instability of glutamine in water, and its easy decomposition into toxic pyroglutamate and ammonia at high temperature, these characteristics limit its wide application in animal feed. Glutamine dipeptide is a dipeptide formed from glutamine and other amino acids (mainly alanine or glycine) that acts physiologically similar to glutamine. Due to its good stability, high water solubility and high utilization efficiency *in vivo*, glutamine dipeptide has been applied to practices related to growth and intestinal immunity in terrestrial (15, 17) and aquatic animals (18–21). For example, glutamine dipeptide can promote the growth of carp (22, 23), increase the weight gain and specific growth rate of hybrid grouper (*Epinephelus fuscoguttatus* ♀ × *Epinephelus lanceolatus* ♂), improve intestinal structure, and decrease the expression of intestinal proinflammatory genes (TNF-α, IL-1β) (24). Butyric acid is a short-chain fatty acid which plays an important role in regulating growth performance, gastrointestinal function, immunity, intestinal pH, and gastrointestinal microecological balance (25). As the main respiratory fuel source of the colonic bacteria, butyrate helps to enhance epithelial cell proliferation and differentiation and improve intestinal absorption function. In addition, many researches have proved that butyrate has potential immunomodulatory and anti-inflammatory properties in the intestine (26). However, butyric acid is very volatile, it is mostly added in the form of sodium butyrate and tributyrin (TB) in feeds. However, sodium butyrate is easy to absorb moisture and agglomerate, and has a special odor, which is not conducive to its wide application. As the precursor of butyric acid, TB can be decomposed by pancreatic lipase in animal intestine to produce 3 molecules of butyric acid and 1 molecule of glycerol, which are then transported by blood to various tissues and organs of the body. The results showed that TB as a feed additive can improve the growth performance of piglets (27), snakehead (*Channa argus*) (28) and yellow catfish (*Pelteobagrus fulvidraco*) (29), and improve the intestinal structures of carps (30), yellow drums (*Nibea albiflora*) (31), and improve digestive enzyme activity and immune performance of juvenile black sea breams (*Acanthopagrus schlegelii*) cultured with high plant protein diet (32).

Largemouth bass, a carnivorous fish native to North America, has been cultivated in many countries, including China, because of its fast growth, delicious meat, and no intermuscular bones. According to statistics, the production of largemouth bass in China had exceeded 700,000 tons in 2021 (China Fishery statistics Year-book, 2022). The commercial feed of largemouth bass typically contains 35%-50% fish meal, which greatly increases feed costs (10). In order to reduce costs, SM is often adopted to replace fish meal. However, replacing fish meal with a high proportion of SM often caused serious negative effects on growth performance, intestinal health and immunity in largemouth bass (33–36). Therefore, there is an urgent need to explore dietary strategies to reduce the negative effects of high percentage of SM on the growth and intestinal health of largemouth bass. In addition, there are few studies on TB and alanyl-glutamine (AGn) related to largemouth bass; in particular, the effect and mechanism on growth and gut health of feeding high soybean meal diet were not clarified (9, 33). Based on the above information, glutamine dipeptide and TB that could provide energy for intestinal epithelial cells and improve intestinal immunity were selected as feed additives in this study. The purpose of this study was to verify the alleviating effects of AGn and TB on intestinal health of largemouth bass cultured with a high proportion of SM by detecting growth performance, intestinal morphology and digestibility, genes related to protein synthesis and inflammation.

## 2 Materials and methods

### 2.1 Experimental diets

A total of 6 diets with isonitrogenous and isolipidic were prepared (37–39). In order to maintain the same animal/plant protein ratio, peanut meal, a plant protein with relatively few anti-nutritional factors and little damage to fish intestinal tract, was selected (40) and the peanut meal group contained 34.80% peanut meal supplemented with methionine, lysine and threonine, which were used as the positive control group (PM); the high SM group with 41.30% SM was supplemented with methionine as the negative control group (SM). With reference to the results of previous studies on TB (41–44) and Ala-Gln (18, 23, 45, 46), 0.10%, 0.20% of TB (group TB0.1 and TB0.2) and 1.00%, 2.00% of Ala-Gln (group AGn1 and AGn2), respectively, were added to SM feed as experimental groups. The feed formula and nutrient content of each experimental group was shown in **Table 1**. Dietary ingredients were ground into fine powder and sieved through a 60-mesh sieve, the ingredients were thoroughly mixed and water was added to form a dough. Then, pellets with 2 mm particle size were produced by using a twin screw extruder. The diets were dried in a ventilated oven at 40 °C and stored at -20 °C (3).

**Table 1:** Feed formula and nutrient levels of diets (air-dry basis).

Ingredients	Composition of diet %					
	PM	SM	TB1	TB2	AGn1	AGn2
Fish meal	35.00	35.00	35.00	35.00	35.00	35.00
Soybean meal	0.00	41.30	41.30	41.30	41.30	41.30
Peanut meal	34.80	0.00	0.00	0.00	0.00	0.00
Pregelatinized starch	5.00	5.00	5.00	5.00	5.00	5.00
Soybean oil	0.00	2.00	2.00	2.00	2.00	2.00
Soy lecithin	2.00	2.00	2.00	2.00	2.00	2.00
Fish oil	1.26	2.00	2.00	2.00	2.00	2.00
Ca(H_2_PO_4_)_2_	2.05	2.00	2.00	2.00	2.00	2.00
Met	1.12	1.20	1.20	1.20	1.20	1.20
Lys	0.13	0.00	0.00	0.00	0.00	0.00
Thr	0.10	0.00	0.00	0.00	0.00	0.00
Vitamin premix^a^	0.50	0.50	0.50	0.50	0.50	0.50
Mineral premix^b^	0.20	0.20	0.20	0.20	0.20	0.20
Choline chloride	0.50	0.50	0.50	0.50	0.50	0.50
MgSO_4_	0.30	0.30	0.30	0.30	0.30	0.30
Microcrystalline cellulose	11.04	2.00	1.90	1.80	1.00	0.00
Carboxymethyl cellulose	3.00	3.00	3.00	3.00	3.00	3.00
Yeast hydrolysate	3.00	3.00	3.00	3.00	3.00	3.00
Tributyrin	0.00	0.00	0.10	0.20	0.00	0.00
Alanyl-glutamine	0.00	0.00	0.00	0.00	1.00	2.00
Total	100.00	100.00	100.00	100.00	100.00	100.00
Nutrient levels						
Crude protein	40.84	40.36	40.73	40.74	40.63	40.43
Crude lipid	9.28	9.88	9.53	9.46	9.58	9.47

^a^Vitamin premix provided the following per kg of diets: VA 2000 IU, VC 175.00 mg, VD_3_ 1000 IU, VE 100.00 mg, VK_3_ 4.80 mg, VB_1_ 14.70 mg, VB_2_ 28.00 mg, VB_6_ 19.60 mg, VB_12_ 0.07 mg, nicotinamide 78.40 mg, D-biotin 0.07 mg, inositol 122.50 mg, folic acid 0.98 mg, D-calcium pantothenate 22.50 mg; 2. ^b^Mineral premix provided the following per kg of diets: MnSO_4_·H_2_O 179.00 mg, CoCl_2_ 1.50 mg, FeSO_4_·7H_2_O 66.00 mg, CuSO_4_·5H_2_O 8.06 mg, ZnSO_4_·H_2_O 150.80 mg, Na_2_SeO_3_ 0.35 mg, KI 6.05 mg.

### 2.2 Feeding and management

Largemouth bass for the experiment was purchased from a farm in Huzhou, Zhejiang of China, and were domesticated in the circulating water system for 2 weeks. At the beginning of the feeding trial, healthy fish with an average body weight of 25.5 ± 0.5 g were randomly distributed to 18 tanks (300 L water), with three replicate tanks for each treatment group and 20 fish per tank. Fish was fed twice daily to apparent satiation at 7 a.m. and 6 p.m. for 8 weeks. During the experiment, one-third of the water was changed every day to maintain water quality. Water temperature, pH, dissolved oxygen, ammonia nitrogen and nitrite were monitored daily and recorded as 27°C to 30°C, above 6.5 mg/L, and below 1.0 mg/L respectively.

### 2.3 Growth performance

At the end of feeding trial, all fish were counted and weighed. Growth performance was determined by calculating the survival rate (SR), weight gain (WG), specific growth rate (SGR), feed coefficient rate (FCR), hepatosomatic index (HSI) and viscerosomatic index (VSI), as follows:


SR(%)=100×(number of final fish/number of initial fish);

WG(%)=100×(final body weight-initial body weight)/initial body weight;

SGR(%/day)=100×(Ln final body weight−Ln initial body weight)/Feeding days;

FCR=feed intake/weight gain;

HIS(%)=100×(final liver weight/final body weight);

VSI(%)=100×(final visceral weight/final weight).

### 2.4 Sample collection

Then, the fish were anesthetized with tricaine methanesulfonate (MS-222), five fish from each tank were randomly selected and stored at -20°C for whole body proximate composition analysis. Three fish from each tank were individually measured body weight, body length, visceral weight, liver weight, to calculate hepatosomatic index (HSI) and viscerosomatic index (VSI). After the dissection, the intestinal tissues were cut into three parts (fore‐, mid‐ and hindgut) and part was stored at −80°C for the digestive enzyme activity analysis, and part was washed with 75% normal saline, foregut, midgut and hindgut were quickly gathered from three fish per tank, then transferred to 4% polyformaldehyde (PFA) fixative for histology structure analysis. Then the whole intestinal tissues of another six fish from each tank were quickly removed and frozen in liquid nitrogen and stored at -80°C for gene expression analysis.

### 2.5 Proximate composition analysis

The moisture, crude protein, crude lipid and crude ash contents were analyzed according to the method of the Association of Official Analytical Chemists (47). The moisture content was determined by drying samples in an oven at 105 °C. The crude protein content was determined using the combustion method by the Dumas nitrogen determination apparatus. The crude lipid content was measured using the ether method in the Soxhlet extraction system. The crude ash was determined, and heated sample was carbonized to smokeless and transferred to a muffle furnace at 550°C for 6 h.

### 2.6 Intestinal histology analysis

Intestinal samples were fixed in the 4% polyformaldehyde (PFA) fixative for 24 h, and then dehydrated in a series of alcohol solutions and embedded in paraffin. The sliced sections (5 μm) of each sample were stained with hematoxylin-eosin (H&E) (Hangzhou Haoke Biotechnology Co., Ltd., Hangzhou, China). The tissue sections were examined using light microscopy with Image-Pro Plus 6 soft-ware (Media Cybernetics, Maryland, USA). It was observed and randomly selected 3 fields of view to take pictures. Villus height (VH), villus width (VW) and muscular thickness (MT) were measured.

### 2.7 Enzyme activity analysis

The frozen guts were thawed at 4 °C, then homogenized with 9 times saline and then centrifuged at 4000 rpm for 10 min (4 °C). The supernatants were collected to measure enzyme activities within 24 h. The trypsin, lipase, α-amylase activities of the gut were tested using the kits (Nanjing Jiancheng Bioengineering Institute) according to the manufacturer’s instructions.

### 2.8 Gene expression analysis

Total RNA was extracted from the whole intestinal tissue using RNAiso Plus (Takara Biotech, Dalian, China). RNA concentration and purity were measured by the spectrophotometry analysis (A260:A280 nm ratio) within the ratio specified by the kit (1.8-2.2). The RNA was reverse transcribed into cDNA with a reverse transcription kit (TaKaRa, Japan) and stored at -20°C for qRT-PCR analysis. The online Primer3 was used to design the primers used for qRT-PCR of the required genes. The primer sequences are shown in **Table 2**. Reverse transcription is carried out according to the instructions of TaKaRa RR047A, and real-time fluorescent quantitative PCR is carried out according to the instructions of the TaKaRa RR420A kit. The transcript levels of genes encoding AMP-activated protein kinase α (AMPKα), the target of rapamycin (TOR), 4E-binding proteins (4E-BPs), acetyl-CoA carboxylase (ACC), caspase-3, caspase-8, caspase-9, tumor necrosis factor (TNF), Interleukin-1β (IL-1β) and transforming growth factor-β (TGFβ1 and TGFβ2) were determined. The relative expression levels of target genes were analyzed using the 2^-△△Ct^ comparison Ct value method.

**Table 2:** Primers sequences for qRT-PCR of largemouth bass.

Primer name	Primer sequence	Primer type	Primer ID
AMPK-α	CAGAAACACGAAGGCAGGGT	forward	119901030
	GAGACTGCGGATCTTCTGTCTG	reverse	
TOR	GAGGAGGCAGAGAAAGGCTTC	forward	119905919
	TCCCTCCATGCTGCTGATG	reverse	
4E-BP	GCAGGTCAGACCTCCAGGAG	forward	119914828
	CGGTGTAGTGCTGAACAGAGTCC	reverse	
ACC	ATCCCTCTTTGCCACTGTTG	forward	119896220
	GAGGTGATGTTGCTCGCATA	reverse	
caspase-3	GCTTCATTCGTCTGTGTTC	forward	119898808
	CGAAAAAGTGATGTGAGGTA	reverse	
caspase-8	GAGACAGACAGCAGACAACCA	forward	119902447
	TTCCATTTCAGAAAACACATC	reverse	
caspase-9	CTGGAATGCCTTCAGGAGACGGG	forward	119915381
	GGGAGGGGCAAGACAACAGGGTG	reverse	
TNF-α	CTTCGTCTACAGCCAGGCATCG	forward	119897144
	TTTGGCACACCGACCTCACC	reverse	
IL-1β	TGATGAGGGACTGGACCTGG	forward	119914255
	ACCAGGCTGTCCATGATCATG	reverse	
TGFβ1	AGCGCATTGAGGCCATTAG	forward	119882881
	GATGTCTGGTGGGCTCTCG	reverse	
TGFβ2	TGTCGTCCGTGTTCTCCTCAC	forward	119896887
	GTAAATGGCGACAATCTCACG	reverse	
β-actin	GATGGTGGGTATGGGCCAG	forward	119885147
	GAGCCTCTGTGAGCAGGACAG	reverse	

### 2.9 Statistical analysis

The data were analyzed by one-way analysis of variance (ANOVA) using SPSS 25.0 statistical software (IBM Corp., Armonk, NY, USA). Tukey’s test was used for multiple comparisons if the difference between groups was significant (*P*<0.05). Differences were considered significant at *P*< 0.05. All data are expressed as means ± SE.

## 3 Results

### 3.1 Growth performance and body composition of fish

As shown in **Table 3**, there were no significant differences in SR, WG, SGR and HSI among all groups (*P*>0.05). And there were no significant differences in FCR between SM group and PM group, while FCR in AGn1, AGn2 and TB0.2 groups was significantly lower than that in SM group (*P*<0.05). Meanwhile, the VSI of AGn2 group was significantly lower than that of PM and SM groups (*P*<0.05). As shown in **Table 4**, there were no significant differences in crude protein, crude lipid, crude ash and moisture among all groups (*P*>0.05).

**Table 3:** Growth performance of largemouth bass fed with different diets.

Groups	SR (%)	WG (%)	SGR (%/d)	FCR	HSI (%)	VSI (%)
PM	76.67 ± 10.14	185.51 ± 9.80	1.87 ± 0.06	1.17 ± 0.12^ab^	1.71 ± 0.17	7.56 ± 0.18^a^
SM	80.00 ± 15.28	179.85 ± 7.95	1.84 ± 0.05	1.30 ± 0.13^a^	1.85 ± 0.06	7.89 ± 0.21^a^
AGn1	93.33 ± 4.41	189.05 ± 16.49	1.89 ± 0.10	0.99 ± 0.03^b^	1.54 ± 0.07	7.77 ± 0.54^ab^
AGn2	100.00 ± 0.00	212.70 ± 11.58	2.00 ± 0.04	0.88 ± 0.08^b^	1.05 ± 0.26	6.46 ± 0.49^b^
TB0.1	80.00 ± 17.56	164.22 ± 14.43	1.73 ± 0.10	1.61 ± 0.32^a^	1.87 ± 0.08	8.24 ± 0.15^a^
TB0.2	86.67 ± 10.93	185.28 ± 14.73	1.87 ± 0.09	0.90 ± 0.06^b^	1.55 ± 0.07	7.31 ± 0.52^ab^

Survival rate (SR), weight gain (WG), specific growth rate (SGR), feed coefficient rate (FCR), hepatosomatic index (HSI), viscerosomatic index (VSI). The value is expressed as mean ± SE. There were significant differences in the data within the parameters of each column with different lowercase letters (P< 0.05).

**Table 4:** Composition of largemouth bass fed with different diets.

Groups	Crude protein %	Crude lipid %	Crude ash %	Moisture %
PM	17.24 ± 0.03	4.48 ± 0.08	3.86 ± 0.03	70.59 ± 0.83
SM	17.01 ± 0.05	4.62 ± 0.05	3.89 ± 0.04	70.81 ± 0.28
AGn1	17.00 ± 0.09	4.51 ± 0.05	3.89 ± 0.04	70.46 ± 0.66
AGn2	17.09 ± 0.11	4.55 ± 0.06	3.95 ± 0.02	70.50 ± 0.60
TB1	16.96 ± 0.08	4.65 ± 0.06	3.88 ± 0.03	71.05 ± 0.46
TB2	16.95 ± 0.06	4.64 ± 0.06	3.87 ± 0.04	70.65 ± 0.68

The value is expressed as mean ± SE. There were significant differences in the data within the parameters of each column with different lowercase letters (P<0.05).

### 3.2 Digestive enzymes activity in the intestine

**Figure 1:**
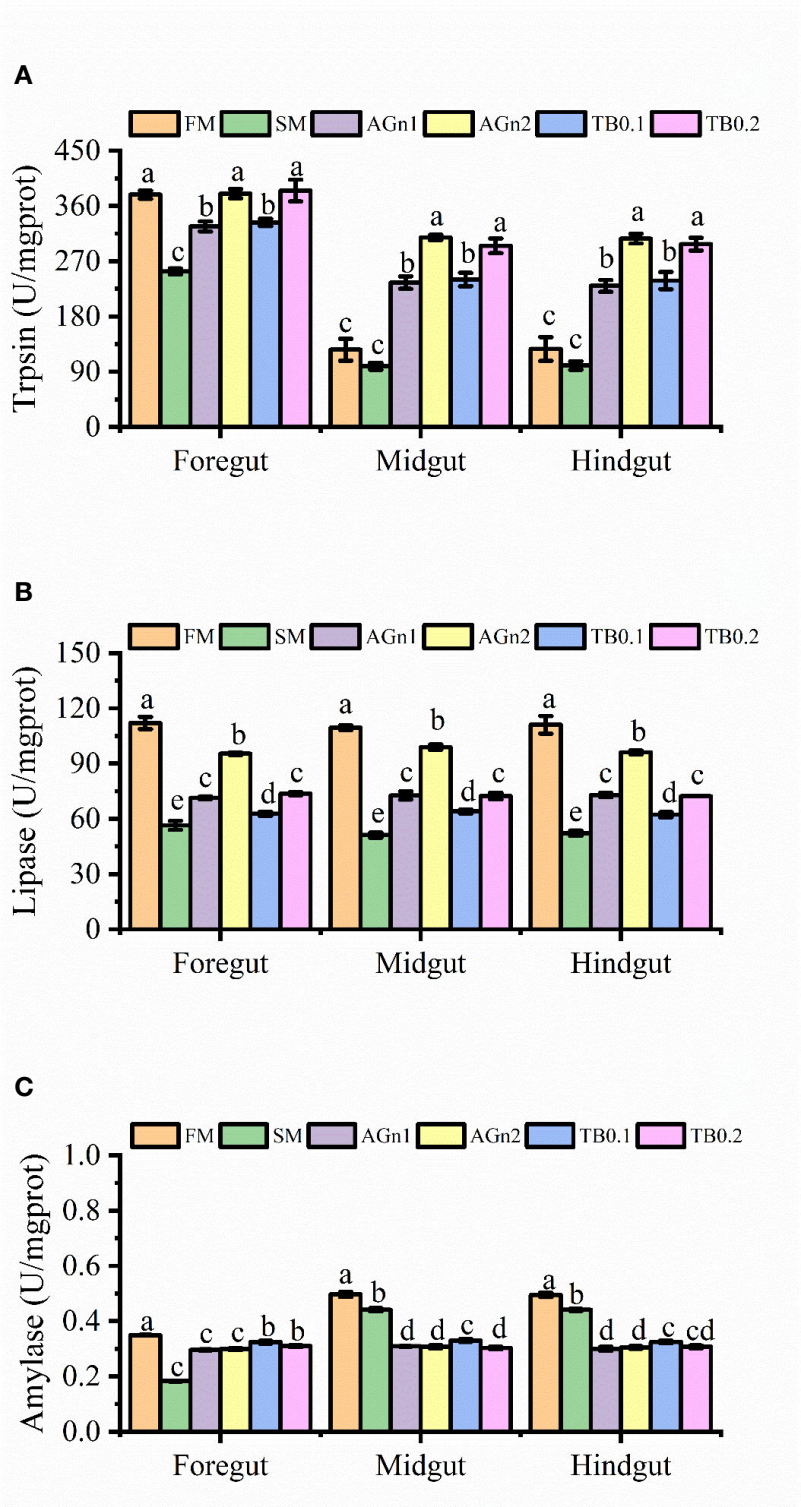
The results of digestive enzyme activities **(A)**, trypsin; **(B)**, Lipase; **(C)**, amylase) in fore-, mid- and hindgut of largemouth bass fed with different diets. Data are shown as mean ± SE. Value with different superscripts are significantly different (*P*<0.05).

As shown in **Figure 1**, compared with the PM group, the activities of lipase and amylase in the foregut, midgut and hindgut, trypsin in the foregut of largemouth bass decreased significantly in SM group (*P*<0.05). Compared with SM group, lipase and trypsin in the whole intestine increased significantly by the addition of high or low dose of the two additives (*P*< 0.05 or *P*<0.01), and the activities of high dose groups were greater than those of low dose groups (*P*<0.05). In terms of amylase, compared with SM group, both additives significantly increased the activity of amylase in the foregut (*P*<0.05), but decreased the activity of amylase in the midgut and hindgut (*P*<0.05), and there were no significant differences in high dose and low dose groups except activities in TB of the midgut (*P*>0.05).

### 3.3 Intestinal morphology

**Figure 2:**
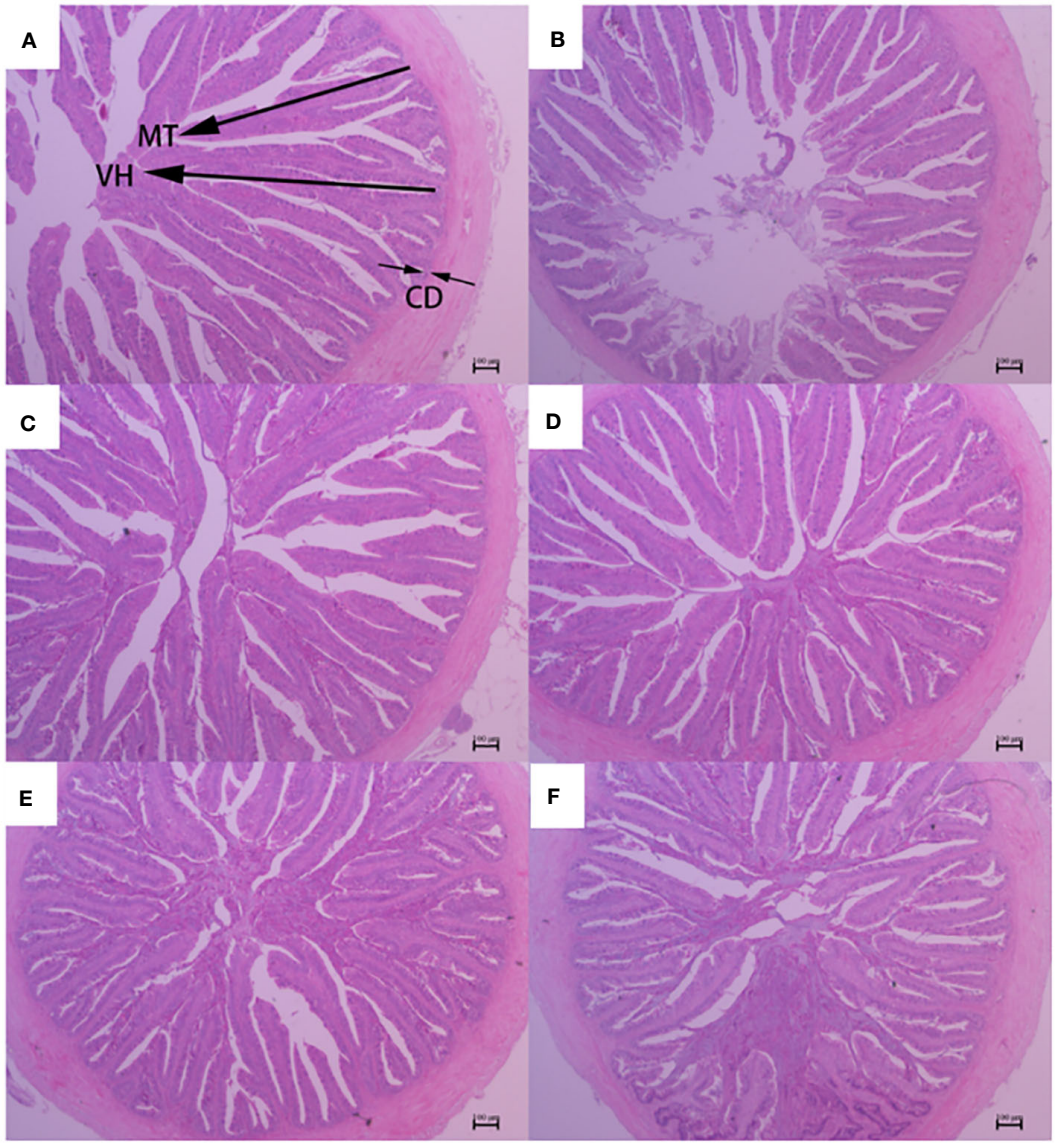
Foregut morphology of largemouth bass fed diets containing different levels of AGn and TB for 8 weeks. **(A)**; PM group, **(B)**; SM group, **(C)**; AGn1 group, **(D)**; AGn2 group, **(E)**; TB0.1 group, **(F)**; TB0.2 group. VH, villus height, CD, crypt depth, MT, mucosal thickness. (HE staining, original magnification ×40). The arrows indicate the positions of MT (mucosal thickness), VH (villus height) and CD (crypt depth).

**Figure 3:**
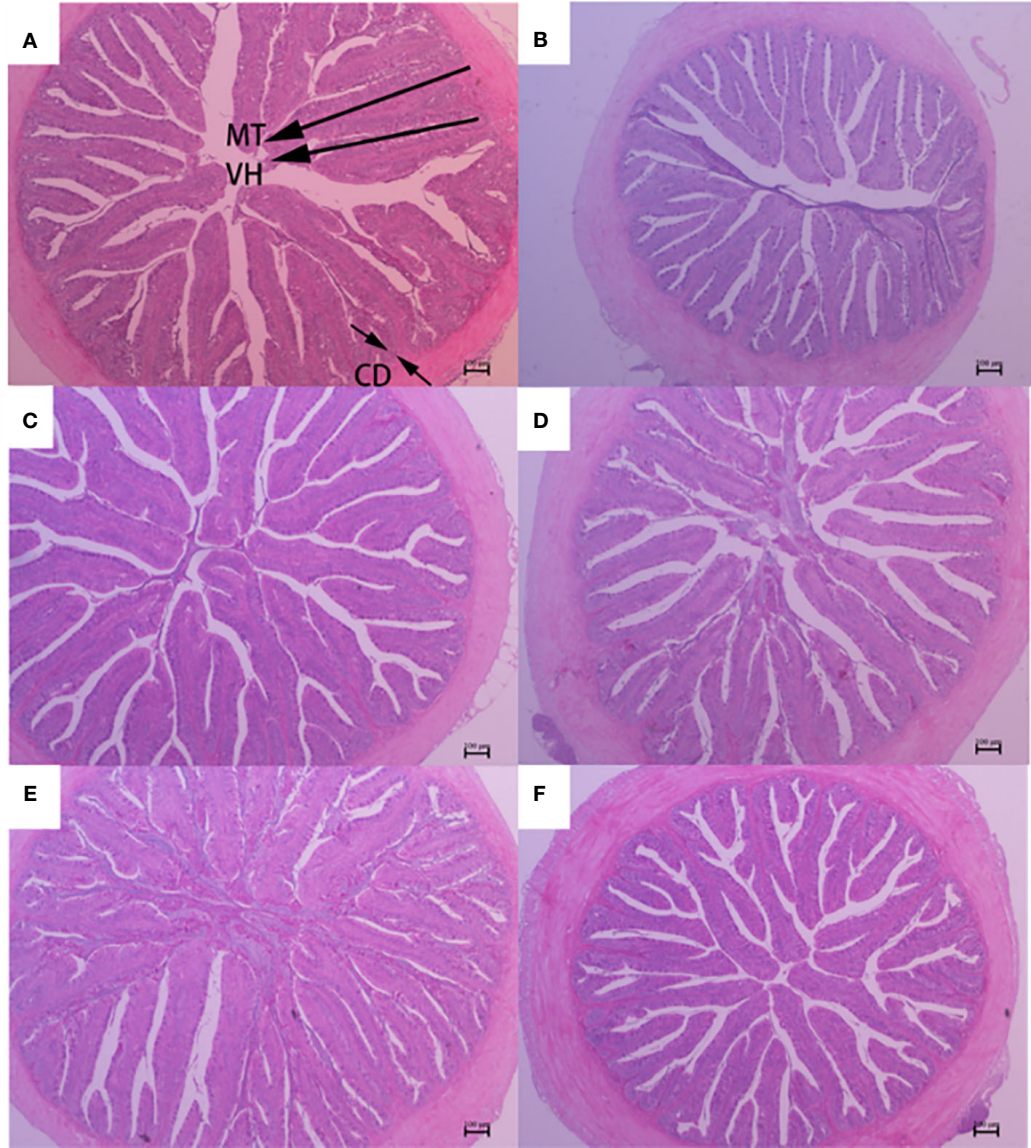
Midgut morphology of largemouth bass fed diets containing different levels of AGn and TB for 8 weeks. **(A)**; PM group, **(B)**; SM group, **(C)**; AGn1 group, **(D)**; AGn2 group, **(E)**; TB0.1 group, **(F)**; TB0.2 group. VH, villus height, CD, crypt depth, MT, mucosal thickness. (HE staining, original magnification ×40). The arrows indicate the positions of MT (mucosal thickness), VH (villus height) and CD (crypt depth).

**Figure 4:**
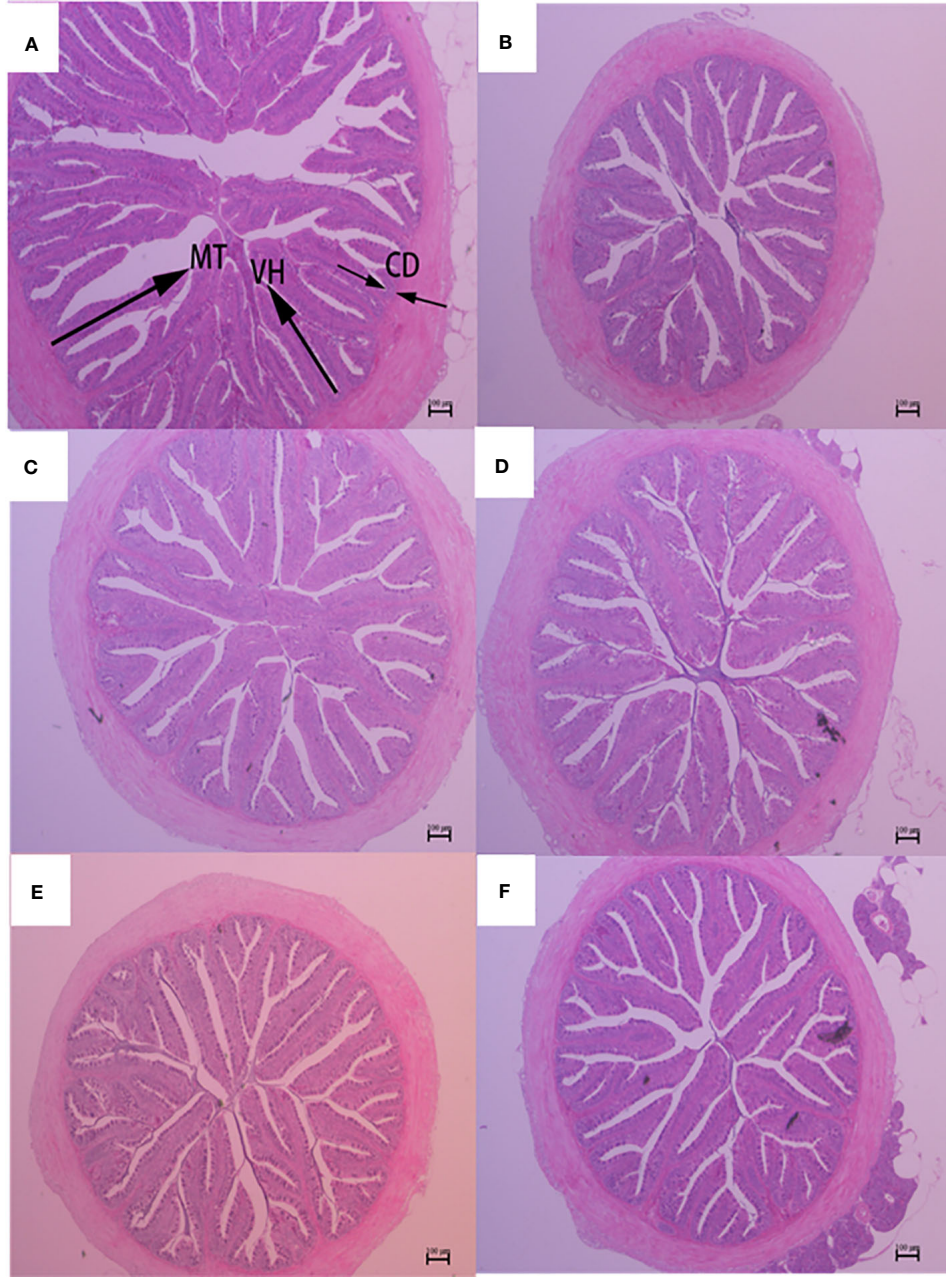
Hindgut morphology of largemouth bass fed diets containing different levels of AGn and TB for 8 weeks. **(A)**; PM group, **(B)**; SM group, **(C)**; AGn1 group, **(D)**; AGn2 group, **(E)**; TB0.1 group, **(F)**; TB0.2 group. VH, villus height, CD, crypt depth, MT, mucosal thickness. (HE staining, original magnification ×40). The arrows indicate the positions of MT (mucosal thickness), VH (villus height) and CD (crypt depth).

The histological features of the foregut, midgut, and hindgut of largemouth bass were presented in **Figures 2**–**4**, and the villus height (VH), crypt depth (CD), and mucosal thickness (MT) were shown in **Table 5**. There were cleavage and necrosis at the top of villus in the foregut of SM group. Compared with the PM group, VH and MT decreased significantly in the foregut, midgut, and hindgut of SM group (*P*<0.05), while CD increased significantly in the foregut (*P*<0.05) rather than in the midgut and hindgut. Compared with SM group, VH and MT in the foregut, midgut, and hindgut in the AGn and TB groups increased significantly (*P*<0.05). In the foregut, in terms of VH and MT, they were obviously higher in AGn groups than those in TB groups (*P*<0.05), and in AGn1 group they are higher than those in AGn2 group (*P*<0.05), but there were no significant differences between TB0.1 and TB0.2 (*P*>0.05). In the midgut, VH and MT in AGn2 group were significantly higher (*P*<0.05) and AGn1 were significantly lower (*P*<0.05) than those in TB0.1 and TB0.2 groups, and there was no significant difference between TB0.1 and TB0.2 (*P*>0.05). In the hindgut, there was no significant difference in VH and MT among the two additives and different concentration (*P*>0.05). Compared with PM group, CD in SM group increased significantly in the foregut, but no significant changes in the midgut and hindgut. Compared with SM group, CD in the foregut, midgut, and hindgut reduced significantly in different concentrations of the two additives (*P*<0.05) except AGn1 in hindgut. In the foregut, CD in TB2 group was significantly lower than that in AGn1, AGn2 and TB1, but no obvious differences were found between AGn1 and AGn2 groups. In the midgut, CD in AGn groups were obviously lower than those in TB groups, but no obvious differences between the two doses of AGn or TB. In the hindgut, CD in the high dose groups were obviously lower than those in low dose groups of the two additives, but no obvious differences between the two low dose groups.

**Table 5:** Intestine morphology indexes of largemouth bass fed with different diets.

Tissues	Groups	Villus height (μm)	Crypt depth (μm)	Mucosal thickness (μm)
Foregut	PM	1015.58 ± 1.11^c^	30.66 ± 0.96^b^	1049.49 ± 0.92^b^
	SM	871.04 ± 18.98^e^	41.60 ± 2.32^a^	915.59 ± 16.71^d^
	AGn1	1037.50 ± 2.28^a^	28.20 ± 0.80^b^	1068.34 ± 2.60^a^
	AGn2	1020.00 ± 1.62^b^	26.28 ± 1.03^b^	1048.83 ± 0.91^b^
	TB0.1	984.83 ± 4.73^d^	25.82 ± 0.64^b^	1013.56 ± 4.51^c^
	TB0.2	953.03 ± 8.54^d^	21.01 ± 0.95^c^	977.75 ± 8.96^c^
				
Midgut	PM	855.50 ± 21.58^b^	47.06 ± 1.37^a^	900.77 ± 21.57^b^
	SM	557.82 ± 31.01^d^	42.69 ± 0.87^a^	598.25 ± 31.37^d^
	AGn1	765.88 ± 5.49^c^	30.37 ± 1.32^c^	798.91 ± 6.36^c^
	AGn2	1001.75 ± 24.60^a^	25.11 ± 1.90^c^	1052.34 ± 25.43^a^
	TB0.1	857.00 ± 6.54^b^	38.16 ± 0.76^b^	898.38 ± 6.21^b^
	TB0.2	855.89 ± 6.37^b^	36.71 ± 1.06^b^	896.37 ± 5.96^b^
				
Hindgut	PM	547.99 ± 1.66^b^	51.52 ± 2.93^a^	578.56 ± 0.97^b^
	SM	509.46 ± 4.66^c^	53.25 ± 3.13^a^	559.31 ± 2.50^c^
	AGn1	654.64 ± 13.42^a^	47.90 ± 2.54^a^	705.28 ± 15.93^a^
	AGn2	734.28 ± 23.55^a^	27.47 ± 1.05^c^	788.92 ± 26.38^a^
	TB0.1	730.90 ± 22.97^a^	34.47 ± 1.29^b^	768.20 ± 23.79^a^
	TB0.2	673.45 ± 16.02^a^	25.11 ± 1.90^c^	729.37 ± 18.94^a^

The value is expressed as mean ± SE. There were significant differences in the data within the parameters of each column with different lowercase letters (P<0.05).

### 3.4 Genes expression of intestinal immunity

**Figure 5:**
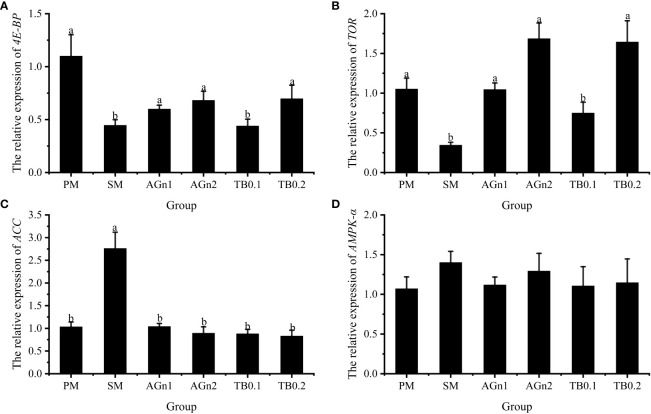
Effects of different treatments on the mRNA levels of 4E-BP **(A)**, TOR **(B)**, ACC **(C)** and AMPK-α **(D)** in intestines of largemouth bass. Data are shown as mean ± SE. Value with different superscripts are significantly different (P< 0.05).

**Figure 6:**
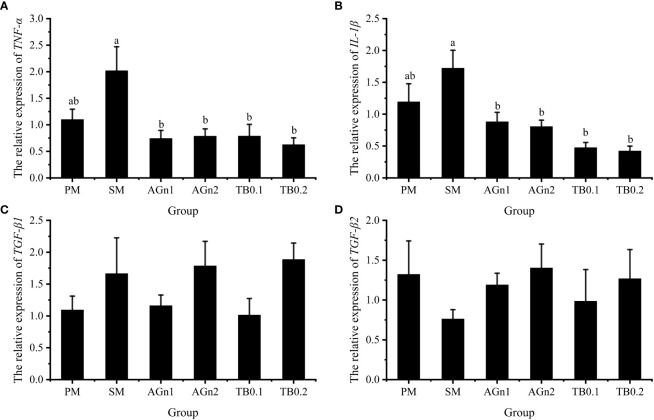
Effects of different treatments on the mRNA levels of TNF-α **(A)**, IL-1β **(B)**, TGFβ1 **(C)** and TGFβ2 **(D)** in intestines of largemouth bass. Data are shown as mean ± SE. Value with different superscripts are significantly different (P<0.05).

**Figure 7:**
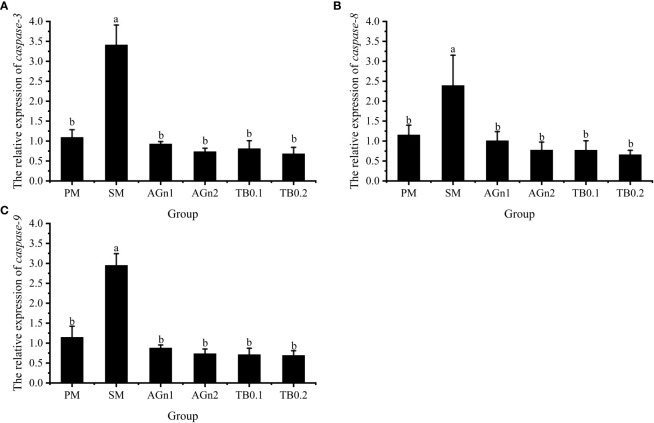
Effects of different treatments on the mRNA levels of caspase3 **(A)**, caspase8 **(B)** and caspase9 **(C)** in intestines of largemouth bass. Data are shown as mean ± SE. Value with different superscripts are significantly different (P<0.05).

The expression of metabolism and immune related genes in the intestine of largemouth bass was shown in **Figures 5**, **6**. Specifically, mRNA expression levels of TOR and 4E-BP in SM group were significantly lower than those in PM group (*P*<0.05). (**Figures 5A, B**). However, the expressions of ACC, caspase, TNF-α, and IL-1β showed opposite trend (*P<* 0.05) (**Figure 5C**, **Figures 7A–C**, **Figures 6A, B**). Compared with SM group, higher expressions of TOR and 4E-BP were observed in the experimental groups (AGn and TB0.2), while no significant differences were found in TB0.1 group (*P* > 0.05) (**Figures 5A, B**). The mRNA expression levels of ACC, caspase, TNF-α, and IL-1β in the experimental groups were significantly lower than those in SM groups (*P*< 0.05) (**Figure 5C**, **Figure 7**, **Figures 6A, B**). The mRNA expressions of AMPK-α, TGFβ1, and TGF-β2 were not significant different among all experimental groups (*P* > 0.05) (**Figure 5D**, **Figures 6C, D**).

## 4 Discussion

Supplementing butyrate and glutamine has a positive effect on the growth performance of fish fed with plant-based diet (15, 24). It has been reported that TB could enhance the WG of grass carp (*Ctenopharyngodon idellus*) (43, 48), hybrid grouper (49) and blunt snout bream (*Megalobrama amblycephala*) (44), but had no significant effects on SR, FCR, HSI, VSI and body composition of grass carp (43, 48). Dietary glutamine or alanyl-glutamine increased the WG of hybrid grouper (24), Jian carp (50), Pacific white shrimp (*Litopenaeus vannamei*) (21), yellow catfish (20) and largemouth bass (9, 33). However, in this study, two additives had no significant effects on the growth performance and body composition of largemouth bass fed with high soybean meal. These inconsistent research results may be related to the variety and status of experimental animals (51). Interestingly, the FCR of largemouth bass decreased after TB or AGn supplementation and the VSI also decreased after AGn addition, which indicated that both additives improved the feed utilization of fish in our study. Similar results were also found in studies of Ala-glutamine on hybrid grouper (24), *Penaeus vannamei* (21), grass carp (52), and on studies of TB in hybrid grouper (49), blunt snout bream (44), grass carp (43). So, the improved FCR may be due to more energy provided by the two additives for intestinal tract, further improving the health of intestine.

The morphology and structure of animal intestine can affect the absorption and utilization of nutrients as well as the growth and development of animals, among which the thickness of intestinal mucosa, villus height and crypt depth are usually used as indicators of intestinal digestion and absorption capacity (29, 53). Studies have shown that feeding high SM to aquatic animals causes damage to intestinal epithelial cells (6, 54, 55). Due to a variety of anti-nutritional factors contained in plant components, dietary plant proteins usually disrupt the digestibility of fish, resulting in reduced intestinal absorption (30, 33, 41, 51). In this study, the gut of largemouth bass, especially the foregut, was damaged in the fish fed soybean meal, and the supplementation of two additives, especially Ala-glutamine, showed obviously improvement on the intestinal barrier. Similar to the results of this study, intestinal damage occurred in black sea bream cultured by high SM diet, which was manifested as reduced intestinal villi height, disruption and atrophy (32). However, intestinal structure was improved when butyrate glycerides and TB were added to the feed (30, 56). The intestinal villus height of grass carp (43), yellow drum (31), yellow catfish (29) and Mirror carp (26) increased significantly after adding appropriate amount of TB in the diet. Dietary supplementation of glutamine or dipeptide products had positive effects on the mucosal thickness and crypt depth of hybrid grouper (24), turbot (*Scophthalmus maximus*) (57) and grass carp (52). The improvement of intestinal structure and morphology may be due to the fact that TB and AGn are the main energy sources of intestinal epithelial cells (15, 58), which can provide energy for intestinal epithelial cells and stimulate their proliferation and differentiation, especially when the intestinal tract is damaged (34, 59, 60). In addition, the present study showed that AGn was superior to TB in improving the intestinal morphology of largemouth bass, especially in the fore- and midgut, and 2% AGn was the most effective. In another study, by adding 0.6%-0.9% AGn in the high-soybean meal diet, the intestinal villus height of largemouth bass was improved, but growth performance was not improved (33). So, the optimal dose of Ala-glutamine in largemouth bass to improve both intestinal structure and growth needs to be further studied.

The activity of digestive enzymes in fish digestive tract is one of the important indexes reflecting the ability of aquaculture animals to digest nutrients in feed (29). In this experiment, a high proportion of SM decreased the activities of lipase and trypsin in the fore-, mid- and hindgut of largemouth bass, and the supplementation of AGn and TB effectively increased the intestinal activities of the two enzymes. This is consistent with the results of studies which added triglyceride to the diet of black sea bream (32), carp (30), yellow catfish (29) and Pacific white shrimp (61), as well as those which supplementation of glutamine or dipeptide to the diet of grass carp (52), Jian carp (50) to improve intestinal trypsin and lipase. The addition of butyrate products increases the activity of digestive enzymes in fish, which may be related to organic acids, such as butyrate, which can reduce the pH of chyme and thus greatly stimulate the secretion of digestive enzymes (62). As the main energy substance of gut (63), glutamine promotes the proliferation of intestinal mucosal cells and the repair of damaged intestinal epithelial cells; in addition, it may also promote the development of pancreatic tissue, an important organ secreted by digestive enzymes (18). The high SM diet decreased the intestinal amylase activity of largemouth bass, but the amylase activity was only increased in the foregut and decreased in the midgut and hindgut when the dietary AGn or TB was supplemented. This is consistent with the study that amylase activity was enhanced by supplementing TB in the diets of young black bream (41) and snakehead (28), and glutamine dipeptide in the diets of grass carp (52). However, this is contrary to the result of grass carp supplemented TB (43). These differences may be due to the low intestinal amylase activity of fishes, especially carnivorous fishes (64).

In addition to the capacity of digestion and absorption, the intestine is the largest immune organ of fish. Previous studies have shown that the use of SM has caused some intestinal health problems in fish, such as impaired intestinal development, inflammation and oxidative damage, but there are few studies on whether TB and AGn can solve these problems in fish. Antinutritional factors in SM are highly immunogenic (2), and are easy to induce immune response, leading to fish intestinal inflammation (65, 66). Both IL-1β and TNF-α are proinflammatory cytokines, while TGF-β1 and TGF-β2 are anti-inflammatory cytokines (67). During cellular oxidative stress, both TNF-α and IL-1β secretes inflammatory cytokines, causing neutrophil aggregation and activating redox sensitive transcription factors, leading to increased inflammatory response and tissue damage (29, 31, 34). In this experiment, expression levels of TNF-α and IL-1β were upregulated in the SM group, but significantly downregulated after TB/AGn supplementation. This indicated that the high proportion of soybean meal did cause intestinal damage, and TB/AGn could effectively inhibit the inflammatory process caused by high level SM in the intestine of largemouth bass. It is generally believed that the inflammation is caused by the anti-nutritional factors contained in soybean meal (30, 51). Studies in this aspect have been confirmed in the studies of various fish species such as yellow drum (*Nibea albiflora*) (31), common carp (30), black sea bream (56), turbot (68), and hybrid grouper (24). Many studies have shown that dietary supplementation of TB or AGn has a protective effect on intestinal inflammation in both terrestrial (17) and aquatic animals (24, 30). Studies have shown that adding a moderate amount of TB to a high plant protein diet can down-regulate the intestinal proinflammatory factors TNF-α and IL-1β in yellow drum (31) and common carp (30). The suppression of the proinflammatory response by TB or Ala-Gln supplementation in soybean meal-based diets may be related to the improvement of the gut physical barrier, as a developed gut physical barrier which protects the gut from pathogenic microorganisms (33, 49). However, no significant differences between TGF-β1 and TGF-β2 anti-inflammatory cytokines was observed in our study, which may be because the TGF was not sensitive to such responses. Similar results were also found in the studies of yellow drum (31).

Apoptosis plays an important role in maintaining homeostasis in the organism. Intestinal inflammation is usually accompanied by an increase in apoptosis (69). Cysteine protease (caspase) is a key enzyme, that causes cell apoptosis and its apoptosis regulatory pathways, include mitochondrial pathway, death receptor pathway and endoplasmic reticulum pathway. Once this signal transduction pathway is activated, caspase will be activated, followed by apoptosis protease cascade reaction. Previous studies have shown that glutamine attenuated the apoptosis induced by 2,4,6-trinitrobenzene sulfonic acid in the rat colon (70), and intestinal caspase-3 and -9 activities of hybrid grouper were significantly lower in 2% Ala-Gln group (33). This is consistent with the current study, implying that glutamine and Ala-Gln both have a potential apoptosis prevention effect by reducing caspase activities. In the case of tributyrin, studies have shown that adding 0.08% TB to a high plant protein diet can downregulate the apoptosis genes(caspase-2 and caspase-8)in hybrid grouper (49). Similar results were also observed in the current study, these apoptosis-related factors caspase-3, -8 and -9 were significantly upregulated in SM group, but downregulated in the experimental groups. Intestinal inflammation is usually accompanied by an increase in apoptosis (69), so the intestinal damage may be alleviated by the decreased expression of genes related to apoptosis after TB/AGn supplementation. This shows that compared with peanut meal, SM can significantly accelerate the apoptosis of intestinal cells of largemouth bass, and TB and AGn supplementation can alleviate the damage of SM to intestinal cells of largemouth bass by inhibiting mitochondrial apoptosis pathway.

Studies in aquatic animals have shown that similarly to butyric acid, glutamine and its dipeptide can downregulate the mRNA expression of TNF-α and IL-1β in the intestine of hybrid grouper (24) and turbot (68), and effectively inhibit the fish enteritis induced by soybean meal. The nuclear factor NF-κB is an important regulator of gene expressions involved in inflammation. It induces inflammatory cytokines and recruits immune cells *via* translocation to the nucleus (71). Activation of NF-κB may lead to inflammation and hyperpermeability of intestinal epithelial cells. These results demonstrate that dietary glutamine probably enhances the intestinal immune defense to alleviate enteritis by inhibiting the MyD88/NF-κB pathway (24). In addition, medical studies have shown that the therapeutic effects of sodium butyrate and AGn on respiratory tract inflammation are exerted by affecting signaling pathways including AMPK, TOR, NF-κB and STAT3. This revealed that glutamine and its dipeptide and butyrate products may have a similar mechanism of action in the treatment of certain inflammations (17). However, the exact mechanism and the involved signal pathway underlying the anti-inflammatory role of these two supplements in fish with soybean meal induced enteritis are not clear and warrant further investigation. Adenylate activated protein kinase (AMPK), whose activity is regulated by many factors such as body energy status, is known as the “cell energy regulator” (72, 73). AMPK is activated by sensing changes in AMP/ATP and ADP/ATP ratios in cells, and promotes catabolic processes of ATP production by inhibiting the anabolic processes of ATP consumption, thus restoring energy balance (74). Acetyl coenzyme A carboxylase (ACC) is the rate limiting enzyme for fatty acid synthesis, which is the main target protein of AMPK regulating fatty acid oxidation (75, 76). When cells are under stress or energy consumption increases and the body energy level is low, the intracellular AMPK content increases, which reduces ACC activity, thereby reducing fatty acid synthesis and improving fatty acid decomposition to meet the demand for energy (77, 78). In this study, the addition of TB and Ala-Gln to soybean meal diets did not affect AMPK expression. The possible reason was that ATP levels did not significantly change during the experiment, and the changes in AMP/ATP and ADP/ATP were not sufficient to affect AMPK phosphorylation. Compared with SM group, the transcription and expression of ACC in intestine of largemouth bass decreased significantly in PM and experimental groups, which was consistent with the research results of broilers (79) and piglets (80). These results indicated that the intestinal damage of largemouth bass in SM group was more serious than that of PM group, and supplementation of TB and Ala-Gln could significantly reduce the intestinal damage caused by soybean meal. As the target protein of rapamycin, TOR is also one of the target proteins regulated by AMPK. Studies have shown that the regulation of nutrient metabolism in animals is all involved in TORC1, a complex formed by TOR (81, 82). TORC1 regulates the overall translation level and regulates protein synthesis and metabolism through its downstream phosphorylation of S6K1 and 4E-BP1 (82). It has been proved that the addition of glutamine increases protein synthesis by increasing phosphorylated TOR and its main downstream effective substrates S6K1 and 4E-BP1 (74, 83, 84). In this study, the PM, AGn and TB0.2 groups all upregulated the expression of TOR and 4E-BP, indicating that they could help the intestine of largemouth bass to deposit protein.

## 5 Conclusion

To sum up, high proportion of soybean meal induces intestinal inflammation, damages intestinal structure and reduces the activities of digestive enzymes of largemmouth bass. Fortunately, the intestinal inflammation was alleviated by the addition of TB and AGn in the feed. The recommended level is 2% AGn and 0.2% TB. This study helps to promote the application of TB and AGn as functional feed additives in aquatic feed in the aspects of theories and practices.

## Data availability statement

The raw data supporting the conclusions of this article will be made available by the authors, without undue reservation.

## Ethics statement

The animal study was reviewed and approved by Animal Experiment Ethics Committee of Huzhou University. Written informed consent was obtained from the owners for the participation of their animals in this study.

## Author contributions

JZ project administration, writing the original draft preparation, and formal analysis. XY and ZQ, formal analysis and investigation. RZ, experiment design and management. HX, investigation. TW, writing the manuscript and critical revision. All authors contributed to the article and approved the submitted version.
